# Benchmarking knowledge distillation for lightweight pneumonia detection: a multi-seed calibration study on PneumoniaMNIST

**DOI:** 10.1186/s13104-026-07793-8

**Published:** 2026-05-11

**Authors:** Ryan Yi Sheng Neo

**Affiliations:** Independent Researcher, Singapore, Singapore

**Keywords:** Knowledge distillation, Pneumonia detection, Model compression, Domain generalization, Chest X-ray

## Abstract

**Objective:**

To benchmark a very small convolutional neural network trained with and without knowledge distillation for pneumonia detection on PneumoniaMNIST, with emphasis on discrimination, calibration, efficiency, and external generalization.

**Results:**

Using the predefined MedMNIST v2 splits and 5 random seeds, a ResNet-18 teacher achieved an area under the receiver operating characteristic curve of 0.9735 (95% confidence interval 0.9687−0.9782). A 60,642-parameter TinyCNN retained about 90% of teacher discrimination (0.8818, 0.8634−0.9001) while reducing parameters by 184-fold, multiply-accumulate operations by 10.9-fold, and batch-1 CPU inference time by 3.7-fold. Knowledge distillation did not provide consistent discrimination gains over vanilla training on this benchmark. The teacher was less well calibrated before temperature scaling, which improved its negative log-likelihood, Brier score, and expected calibration error. External zero-shot evaluation on Kermany and a balanced RSNA subset showed domain-shift degradation for all models, with partial recovery after brief fine-tuning but persistent teacher–student gaps.

## Introduction

Pneumonia remains a major cause of morbidity and mortality worldwide, and chest radiographs are a common screening modality. Deep learning models can assist triage, but many high-performing architectures are too heavy for low-cost, offline inference. Knowledge distillation (KD) transfers predictive behavior from a large teacher to a smaller student and is widely used for model compression [1, 2].

However, clinical deployment also depends on *probability trustworthiness*: overconfident probabilities can lead to unsafe thresholds and miscalibrated risk estimates. Temperature scaling (TS) is a standard post-hoc method for improving calibration without changing discrimination [3].

This research note provides a rigorous multi-seed evaluation of knowledge distillation for pneumonia detection model compression, with emphasis on both discrimination and calibration metrics to inform practitioners’ method selection. We study a practical benchmark question: can a very small student CNN achieve near-teacher performance on PneumoniaMNIST while producing reliable probabilities? To strengthen reproducibility, we (i) report multi-seed confidence intervals and paired tests, (ii) compare response-based KD to a feature-based KD baseline (FitNets), (iii) include calibration across seeds, and (iv) provide qualitative interpretability via Grad-CAM.

## Related work

KD was popularized by Hinton et al. [1] and extended to intermediate-feature distillation such as FitNets [2]. Calibration of neural networks is increasingly recognized as critical for medical decision support; TS is a widely used post-hoc calibrator [3].

In medical imaging, recent work explores KD to compress models for resource-limited hardware, including chest X-ray classification and on-device inference [4–6]. A recent survey summarizes KD design choices and common evaluation pitfalls in medical imaging [7]. Because pneumonia models can fail to generalize across institutions and acquisition protocols, we emphasize external evaluation under domain shift [8].

PneumoniaMNIST is widely used as a lightweight benchmark within the MedMNIST ecosystem, with baseline results for standard architectures reported in the MedMNIST v2 benchmark release [9]. More recent benchmarking work also evaluates compact models and compression methods across MedMNIST tasks (including PneumoniaMNIST) [10, 11]. In contrast, we focus on knowledge distillation and probability calibration with multi-seed statistical reporting and external validation.

## Methods

### Dataset and preprocessing

We use PneumoniaMNIST from MedMNIST v2 [9], a pediatric chest X-ray benchmark with predefined train/val/test splits. Images are grayscale; we replicate channels to RGB, resize to $224\times 224$, and normalize using ImageNet mean/std for compatibility with standard CNN backbones.

### Models

#### Teacher

 ResNet-18 configured for binary classification.

#### Student (TinyCNN)

 A compact CNN with four convolutional blocks, global average pooling, and a linear classifier. From the released checkpoint tensors, TinyCNN has 60,642 trainable parameters (0.231 MB in float32).

### Knowledge distillation objectives

Let $z_t$ and $z_s$ denote teacher and student logits, and $p(\cdot ;T)$ denote softmax with temperature *T*.

#### Response-based KD (logit matching)

1 $$\begin{aligned} & L_{\text {resp}} = \alpha \,\mathcal {L}_{CE}(y, p(z_s;1)) + (1-\alpha )\,T^2\,\nonumber \\ & \quad \textrm{KL}\big (p(z_t;T)\,\Vert \,p(z_s;T)\big ). \end{aligned}$$

#### Feature-based KD (FitNets-style)

 We additionally match pooled intermediate features using mean squared error (MSE) with a learned projection from teacher feature dimension to student feature dimension:

2 $$\begin{aligned} L_{\text {fit}} = \mathcal {L}_{CE}(y,p(z_s;1)) + \beta \,\Vert \phi _s - g(\phi _t)\Vert ^2_2. \end{aligned}$$

The projection $g(\cdot )$ is a single linear layer (no activation) mapping the teacher’s pooled feature dimension (512 for ResNet-18 after adaptive average pooling) to the student’s pooled feature dimension (1024 for TinyCNN: 64 channels $\times$ 4$\times$4 spatial after AdaptiveAvgPool2d). This introduces $512\times 1024 = 524{,}288$ additional trainable parameters during training, which are discarded at inference.

### Training details

All experiments were run in Kaggle on a Tesla T4 GPU. We report results across seeds 0–4.

- Optimizer: Adam ($\beta _1{=}0.9$, $\beta _2{=}0.999$), weight decay $10^{-4}$.
- Teacher: 8 epochs, learning rate $3\times 10^{-4}$, cosine annealing schedule.
- Students (vanilla and KD): 12 epochs, learning rate $5\times 10^{-4}$.
- Batch size: 128 (train), 256 (val/test).
- Augmentation: none beyond resizing and normalization.
- KD selection: a $3\times 3$ sweep on seed 0 over $T\in \{1,2,4\}$ and $\alpha \in \{0.1,0.3,0.5\}$; best configuration used for multi-seed runs was $(T,\alpha )=(2,0.3)$. For FitNets, we swept $\beta \in \{10^{-4}, 3\times 10^{-4}, 10^{-3}\}$ and selected $\beta =3\times 10^{-4}$.

We acknowledge that selecting hyperparameters on a single seed is a limitation; ideally, hyperparameters would be validated across multiple seeds. However, given that KD showed no consistent benefits in our multi-seed evaluation, hyperparameter optimization is unlikely to change the core conclusion. To further support this, we note that the selected configuration $(T,\alpha )=(2,0.3)$ is not at an extreme of the swept range: configurations with stronger KD weighting ($\alpha =0.5$) and softer targets ($T=4$) were evaluated on seed 0 but did not outperform the selected setting. The fact that increasing the KD signal strength did not help on seed 0 is consistent with the null multi-seed finding, and suggests the absence of KD benefit reflects the benchmark and capacity regime rather than an under-explored hyperparameter space. Similarly, the FitNets $\beta$ sweep covered a 10-fold range ($10^{-4}$ to $10^{-3}$), with the intermediate value selected; the null finding persists across all seeds for this variant as well.

Epoch counts were chosen to allow models to converge while maintaining computational feasibility. We did not implement formal early stopping.

All 95% confidence intervals are computed as mean ± (*t*-critical $\times$ SEM), where SEM is the standard error of the mean across 5 seeds and *t*-critical is from Student’s *t*-distribution with 4 degrees of freedom.

### External evaluation and fine-tuning

#### Zero-shot

 We evaluate the trained PneumoniaMNIST models on two external CXR datasets without adapting weights: (i) the Guangzhou pediatric CXR dataset (“Kermany”) [12], and (ii) a balanced subset of the RSNA Pneumonia Detection Challenge [13]. We reuse the same preprocessing (grayscale $\rightarrow$ RGB, $224\times 224$ resizing, ImageNet normalization) and report mean metrics with 95% CIs across 5 seeds. We emphasize that the RSNA subset is artificially balanced (50/50 pneumonia/normal) and does not reflect the challenge’s original class distribution or real-world screening prevalence. Results on this subset should be interpreted as a controlled domain-shift probe, not as representative of deployment performance.

#### Brief fine-tuning

 For target-domain adaptation, we replace the final classifier for binary pneumonia prediction and fine-tune using Adam with learning rate $1\times 10^{-4}$ for 2 epochs on the external training split (batch size 64). To reduce overfitting risk given the limited external training data (Kermany: $\sim$5k images; RSNA balanced subset: 400 images), we freeze the ResNet-18 feature extractor and train only its final layer; for TinyCNN variants we fine-tune all layers. We acknowledge this asymmetry creates a confounding factor that may favor TinyCNN in the fine-tuning comparison; however, the alternative of fully fine-tuning ResNet-18 carries high overfitting risk with such limited data. This protocol prioritizes stable comparison over maximizing individual model performance. We select checkpoints by validation AUC and report test metrics. (Kermany results use seeds 0–2; RSNA uses seeds 0–4.)

## Results

### Discrimination performance

Table 1 reports mean test discrimination metrics with 95% CIs of the mean (5 seeds), including AUC and area under the precision–recall curve (AUPRC). TinyCNN retains $\sim$90% of teacher AUC while reducing parameters by $\sim$184$\times$.

**Table 1:** PneumoniaMNIST test performance (mean; 95% CI of mean)

Model	$\textbf{N}$	AUC	AUPRC	Sens@0.5	Spec@0.5
ResNet-18 (teacher)	5	0.9735 [0.9687, 0.9782]	0.9765 [0.9700, 0.9830]	0.9974 [0.9952, 0.9997]	0.7068 [0.6564, 0.7573]
TinyCNN (vanilla)	5	0.8818 [0.8634, 0.9001]	0.9009 [0.8817, 0.9202]	0.9472 [0.9312, 0.9632]	0.5923 [0.5221, 0.6626]
TinyCNN + KD (response)	5	0.8760 [0.8586, 0.8935]	0.8982 [0.8906, 0.9058]	0.9323 [0.9025, 0.9621]	0.5957 [0.5522, 0.6393]
TinyCNN + KD (FitNets)	5	0.8820 [0.8756, 0.8884]	0.8998 [0.8956, 0.9040]	0.9508 [0.9409, 0.9607]	0.5829 [0.5518, 0.6140]

#### Significance testing

 Paired AUC tests across matching seeds show no significant difference between KD variants and vanilla (response-KD vs vanilla: paired *t*-test $p=0.62$, Wilcoxon $p=1.0$; FitNets-KD vs vanilla: paired *t*-test $p=0.97$, Wilcoxon $p=0.81$). We therefore interpret KD as *not providing consistent discrimination gains* on PneumoniaMNIST at this student capacity ($\sim$60k parameters).

### Efficiency

**Fig. 1:**
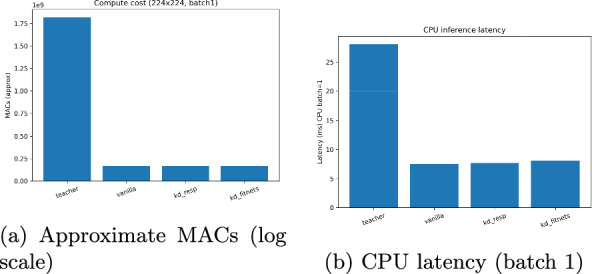
Compute and runtime comparison. TinyCNN reduces MACs by 10.9$\times$ and is 3.7$\times$ faster than the teacher on CPU (batch 1)

Table 2 summarizes parameter counts, approximate MACs/FLOPs, and batch-1 runtime. Figure 1 visualizes the compute and runtime gap between the teacher and TinyCNN. TinyCNN is 0.231 MB (float32 weights), compared with 42.64 MB for the ResNet-18 teacher, and reduces approximate MACs by 10.9$\times$.

**Table 2:** Efficiency metrics (single forward pass, $224\times 224$ input; batch 1)

Model	Params	MB	MACs (M)	FLOPs (G)	CPU ms	GPU ms
ResNet-18 (teacher)	11,177,538	42.64	1813.6	3.63	28.05	2.25
TinyCNN (vanilla)	60,642	0.231	166.2	0.33	7.53	0.45
TinyCNN + KD (response)	60,642	0.231	166.2	0.33	7.67	0.46
TinyCNN + KD (FitNets)	60,642	0.231	166.2	0.33	8.10	0.46

MACs are estimated from layer shapes; FLOPs $\approx 2\times$MACs. Latency measured on Kaggle CPU and Tesla T4 GPU. Latency measurements are indicative only (Kaggle environment with shared resources) and are not representative of dedicated deployment hardware such as edge devices or mobile SoCs

### External validation under domain shift

#### Zero-shot evaluation

To probe generalization beyond PneumoniaMNIST, we evaluate the same trained models *without fine-tuning* on two external chest X-ray datasets using the same preprocessing (grayscale $\rightarrow$ RGB, resize to $224\times 224$, ImageNet normalization). We report mean metrics with 95% CIs across 5 seeds (Table 3).

**Table 3:** Zero-shot external evaluation (mean; 95% CI of mean, 5 seeds)

Model	Kermany AUC	Kermany AUPRC	RSNA AUC	RSNA AUPRC
ResNet-18 (teacher)	0.9547 [0.9520, 0.9573]	0.9717 [0.9692, 0.9741]	0.7458 [0.7262, 0.7655]	0.7007 [0.6774, 0.7240]
TinyCNN (vanilla)	0.8439 [0.8234, 0.8645]	0.8914 [0.8759, 0.9068]	0.6650 [0.6571, 0.6729]	0.6409 [0.6368, 0.6451]
TinyCNN + KD (response)	0.8538 [0.8191, 0.8884]	0.8971 [0.8680, 0.9262]	0.6717 [0.6502, 0.6933]	0.6416 [0.6343, 0.6489]
TinyCNN + KD (FitNets)	0.8437 [0.8345, 0.8529]	0.8891 [0.8815, 0.8966]	0.6657 [0.6537, 0.6777]	0.6430 [0.6384, 0.6477]

Kermany is the Guangzhou pediatric CXR dataset [12]. RSNA is a balanced 1k-image subset (500/500) of the RSNA Pneumonia Detection Challenge [13], sampled deterministically (seed=0) using the provided script. This balanced design does not reflect real-world prevalence

Both student and teacher degrade under domain shift, with the teacher retaining a larger margin. These results reinforce that benchmark performance is insufficient for clinical claims; external validation and/or target-domain fine-tuning are necessary before deployment.

#### Brief target-domain fine-tuning

To test whether modest supervision can reduce domain shift, we fine-tune each model for a small number of epochs on the external training splits and evaluate on the corresponding external test sets (Kermany: 3 seeds; RSNA: 5 seeds; Table 4).

**Table 4:** External fine-tuning results

	Kermany (Guangzhou pediatric CXR) [12]			RSNA subset [13]		
Model	AUC	AUPRC	Spec@0.95	AUC	AUPRC	Spec@0.95
ResNet-18 (teacher)	0.967 ± 0.002	0.978 ± 0.001	0.842 ± 0.000	0.763 ± 0.016	0.732 ± 0.024	0.293 ± 0.016
TinyCNN (vanilla)	0.883 ± 0.032	0.906 ± 0.033	0.584 ± 0.085	0.687 ± 0.012	0.649 ± 0.014	0.118 ± 0.014
TinyCNN + KD (response)	0.888 ± 0.010	0.910 ± 0.010	0.611 ± 0.018	0.688 ± 0.009	0.651 ± 0.010	0.135 ± 0.015
TinyCNN + KD (FitNets)	0.890 ± 0.014	0.912 ± 0.014	0.618 ± 0.028	0.690 ± 0.008	0.654 ± 0.011	0.131 ± 0.012

Kermany uses seeds 0–2 (3 seeds) due to computational constraints, whereas RSNA uses seeds 0–4 (5 seeds). Spec@0.95 is specificity at the most specific threshold achieving sensitivity $\ge 0.95$

### Calibration and high-sensitivity operating point

**Fig. 2:**
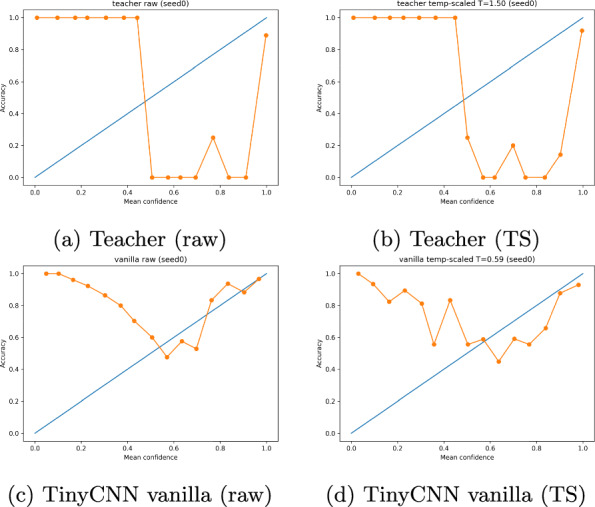
Reliability diagrams (seed 0, test split) before and after temperature scaling. Diagonal is ideal calibration

We evaluate calibration using expected calibration error (ECE), negative log-likelihood (NLL), and Brier score, and apply temperature scaling (TS) [3]. Representative reliability diagrams are shown in Fig. 2. We also report a clinically motivated operating point: choose the *most specific* threshold that achieves sensitivity $\ge 0.95$.

It is important to distinguish probability calibration from discrimination: while the teacher exhibits higher ECE (worse calibration in the probabilistic sense), it achieves substantially higher specificity at the Sens$\ge$0.95 operating point ($\approx$0.91 vs $\approx$0.55-−0.59 for TinyCNN variants), reflecting its superior underlying discriminative capacity. Temperature scaling improves the teacher’s probability estimates without changing this operating-point performance.

Table 5 reports raw (pre-TS) calibration, and Table 6 reports calibration after temperature scaling (TS). Both tables additionally report the resulting specificity at sensitivity $\ge 0.95$. Temperature scaling substantially improves the teacher’s NLL/Brier and reduces its ECE, while changes for the TinyCNN variants are smaller. At the high-sensitivity operating point, the teacher maintains markedly higher specificity ($\approx 0.91$) than the TinyCNN variants ($\approx 0.55$–0.59), underscoring the accuracy–efficiency trade-off when deploying very small models.

ECE is computed using 15 equal-width bins based on the confidence of the predicted class. For each seed, we compute ECE on the test set, then report the mean and 95% CI across the 5 seeds.

**Table 5:** Calibration before temperature scaling (raw probabilities) on the test set (mean; 95% CI of mean, 5 seeds)

Model	ECE$_{raw}$	NLL$_{raw}$	Brier$_{raw}$	Spec@Sens$\ge$0.95 (raw)
ResNet-18 (teacher)	0.0894 [0.0718, 0.1071]	0.4988 [0.3711, 0.6266]	0.0947 [0.0778, 0.1115]	0.9145 [0.8965, 0.9325]
TinyCNN (vanilla)	0.0384 [0.0317, 0.0452]	0.4372 [0.3934, 0.4810]	0.1338 [0.1205, 0.1472]	0.5949 [0.5532, 0.6366]
TinyCNN + KD (response)	0.0727 [0.0558, 0.0896]	0.4985 [0.4484, 0.5485]	0.1409 [0.1281, 0.1538]	0.5547 [0.4780, 0.6314]
TinyCNN + KD (FitNets)	0.0310 [0.0223, 0.0397]	0.4378 [0.4210, 0.4547]	0.1346 [0.1285, 0.1407]	0.5829 [0.5628, 0.6030]

ECE: 15 equal-width bins; confidence of predicted class vs correctness. NLL: probability clipping to [1e-7, 1-1e-7]. Lower is better

**Table 6:** Calibration after temperature scaling (TS) on the test set (mean; 95% CI of mean, 5 seeds)

Model	$T^*$	ECE$_{TS}$	NLL$_{TS}$	Brier$_{TS}$	Spec@Sens$\ge$0.95 (TS)
ResNet-18 (teacher)	1.46 [1.34, 1.58]	0.0745 [0.0593, 0.0897]	0.3646 [0.2978, 0.4313]	0.0893 [0.0743, 0.1043]	0.9145 [0.8965, 0.9325]
TinyCNN (vanilla)	0.74 [0.61, 0.87]	0.0497 [0.0459, 0.0535]	0.4571 [0.4124, 0.5018]	0.1349 [0.1210, 0.1489]	0.5949 [0.5532, 0.6366]
TinyCNN + KD (response)	1.26 [1.11, 1.42]	0.0538 [0.0454, 0.0621]	0.4576 [0.4306, 0.4847]	0.1374 [0.1253, 0.1495]	0.5547 [0.4780, 0.6314]
TinyCNN + KD (FitNets)	0.76 [0.72, 0.80]	0.0524 [0.0472, 0.0575]	0.4604 [0.4432, 0.4776]	0.1366 [0.1299, 0.1432]	0.5829 [0.5628, 0.6030]

$T^*$is fit on the validation split. ECE: 15 equal-width bins; confidence of predicted class vs correctness. NLL: probability clipping to [1e-7, 1-1e-7]. Lower is better

### Interpretability and failure modes

**Fig. 3:**
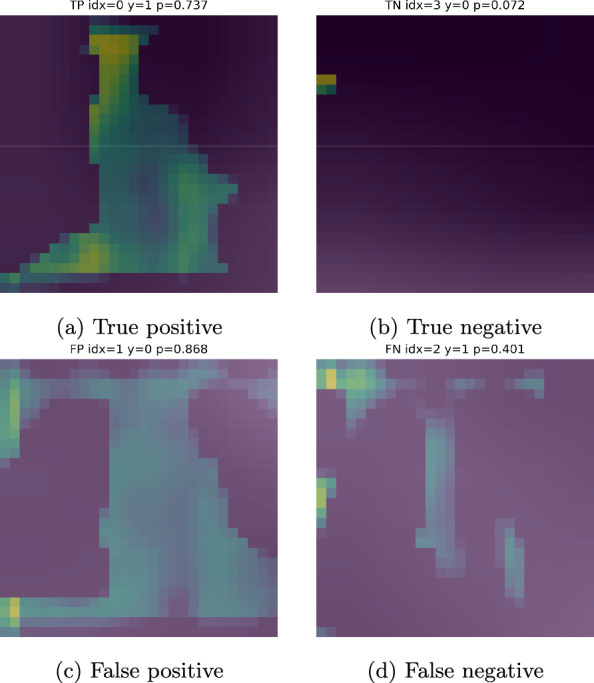
Grad-CAM overlays (seed 0) showing **a** a true positive with attention over a localized lung region, **b** a true negative with more diffuse attention, **c** a false positive where attention is concentrated on non-pathologic structures, and **d** a false negative where the model fails to focus on the abnormal region. Failure cases highlight that attention may be misdirected even when the overall prediction confidence is high. These are qualitative illustrations to provide visual insight into model attention patterns, not a comprehensive explainability assessment

We visualize model attention with Grad-CAM. Figure 3 shows representative true/false positives and negatives. In addition to representative correct cases, we include failure modes (false positive and false negative) to illustrate limitations.

## Discussion

### KD on small benchmarks

 On PneumoniaMNIST, KD did not yield consistent AUC improvements over vanilla TinyCNN. The negative KD findings reported here are specific to PneumoniaMNIST, the chosen TinyCNN capacity ($\sim$60k parameters), and a ResNet-18 teacher that achieves near-saturated performance (AUC 0.9735). One hypothesis is that the ResNet-18 teacher’s high performance may represent a ceiling effect for this relatively simple benchmark, limiting the information content in soft targets. Alternative teacher configurations-such as ensemble teachers, larger architectures (e.g., ResNet-50, EfficientNet), or teachers with higher capacity-might provide richer soft targets. Additionally, testing even smaller student capacities (e.g., <30k parameters) might reveal scenarios where KD becomes more beneficial. These remain open questions for future benchmark-specific investigation. Given the marginal KD effects observed, exhaustive per-seed hyperparameter optimization is unlikely to change the core conclusion. Reporting multi-seed statistics avoids over-interpreting single-seed gains.

### Domain shift and adaptation

 Zero-shot transfer to Kermany and RSNA shows substantial performance degradation for all models, consistent with known dataset and acquisition differences. Several factors likely contribute to this degradation. PneumoniaMNIST (derived from Kermany) and the Kermany dataset itself share the same pediatric source population (Guangzhou Women and Children’s Medical Center), which partly explains the relatively smaller drop on Kermany zero-shot transfer. By contrast, the RSNA dataset covers a broader and predominantly adult patient population, uses different scanner hardware and acquisition protocols, and employs a radiologist opacity-annotation scheme distinct from the binary pneumonia/normal labeling used in PneumoniaMNIST—all of which likely compound the larger RSNA performance drop. Furthermore, PneumoniaMNIST images are downsampled to 28$\times$28 before being resized to $224\times 224$ in our pipeline, which introduces a resolution and texture distribution mismatch not present when directly applying models to full-resolution RSNA images. These differences highlight that domain shift in chest X-ray classification is multifactorial, arising from population demographics, scanner characteristics, image resolution, and annotation protocols simultaneously. Brief fine-tuning on the target-domain training splits recovers part of the drop for TinyCNN variants, but the teacher retains the highest AUC, suggesting that both capacity and pretraining priors matter under distribution shift. The RSNA results use a balanced subset and should not be interpreted as clinically realistic prevalence scenarios. For deployment-oriented studies, we recommend reporting both zero-shot and few-shot external results where possible.

**Calibration in clinical decision support.** Calibrated probabilities are essential for threshold selection and cost-sensitive triage. Across 5 seeds, the teacher has higher ECE than the TinyCNN variants, and temperature scaling improves the teacher’s NLL/Brier and ECE. The TinyCNN variants are already relatively well calibrated and show smaller (and sometimes negligible) benefits from TS. We recommend reporting both discrimination and calibration, and (when relevant) operating-point metrics tied to clinical constraints (e.g., high sensitivity). For clinical deployment, both calibration and operating-point performance matter. The teacher’s higher specificity at high sensitivity is arguably more important than its calibration metrics for triage applications, where ruling out pneumonia at high sensitivity is the primary goal. Calibration becomes critical when actual probability values inform clinical decisions (e.g., risk stratification), not merely binary classification. That said, there are deployment scenarios in which the better-calibrated TinyCNN variants may be preferable despite lower discrimination. When probability outputs are used directly to prioritize a worklist or allocate downstream clinical resources (rather than to produce a binary flag), well-calibrated scores ensure that a model’s stated confidence meaningfully reflects true likelihood of disease. Similarly, in settings where a robust threshold selection procedure is unavailable—for example, when deploying to a new site without access to a calibration holdout set—a model whose raw probabilities are already reliable may be safer to deploy than a higher-discrimination model requiring post-hoc recalibration. Finally, when models are used to communicate uncertainty to clinicians rather than to produce autonomous decisions, the fidelity of the probability estimate itself is the primary requirement, and a small, well-calibrated model may be more appropriate than a larger, overconfident one even at a modest cost in AUC.

## Conclusion and recommendations

On PneumoniaMNIST, a very small convolutional neural network achieves strong benchmark discrimination with substantial efficiency gains, but knowledge distillation does not provide consistent benefits at this student capacity and benchmark difficulty. For deployment-oriented studies, we recommend reporting both discrimination and calibration, evaluating operating points tied to clinical constraints, and testing external generalization under domain shift before making practical claims.

## Limitations

This is a benchmark-focused study, not clinical validation. Limitations include: (i) external evaluation includes both zero-shot and small-split fine-tuning, but RSNA results are reported on a balanced subset rather than the full challenge dataset; (ii) the asymmetric fine-tuning protocol (frozen ResNet-18 backbone vs fully fine-tuned TinyCNN) may underestimate the teacher’s adaptation potential but was necessary to prevent overfitting on small external datasets; (iii) resizing to $224\times 224$ is convenient for standard backbones but not necessarily optimal for edge deployment; and (iv) deployment evidence is limited to approximate MACs and Kaggle CPU/GPU latency. Critically, Kaggle-measured latency should not be taken as representative of real edge-device performance: shared cloud compute does not reflect the memory bandwidth constraints, energy envelopes, thermal throttling, or integer/fixed-point quantization overheads of mobile SoCs, microcontrollers, or other embedded inference hardware. Meaningful deployment characterization would require on-device profiling (e.g., Raspberry Pi, ARM Cortex-M series, or mobile NPU) with energy measurement. Future work should test on external CXR datasets, report FLOPs/activation memory, and evaluate runtime on representative edge hardware.

## Data Availability

PneumoniaMNIST is publicly available through MedMNIST v2 with predefined splits [4]. External datasets used for domain shift experiments are publicly accessible: the Guangzhou pediatric CXR dataset (Kermany) [7] and the RSNA Pneumonia Detection Challenge data [8]; access may require accepting the dataset-specific terms. We provide exact dataset folder expectations and preprocessing code in the accompanying code package so results can be reproduced.

## Appendix A: Per-seed results

Table 7 lists per-seed AUC for reproducibility.

**Table 7:** Per-seed test AUC (seeds 0–4)

Model	0	1	2	3	4
Teacher	0.9733	0.9791	0.9729	0.9684	0.9736
Vanilla	0.8994	0.8684	0.8673	0.8947	0.8790
KD (resp)	0.8803	0.8907	0.8678	0.8560	0.8855
KD (FitNets)	0.8821	0.8807	0.8758	0.8815	0.8901
